# The promotive role of USP1 inhibition in coordinating osteogenic differentiation and fracture healing during nonunion

**DOI:** 10.1186/s13018-023-03594-y

**Published:** 2023-03-02

**Authors:** Jun Huang, Hongxiang Zhou, Liang He, Lin Zhong, Ding Zhou, Zongsheng Yin

**Affiliations:** 1 The Microscopic Repair and Reconstruction Department of Hand and Foot, Department of Orthopedics, the First Affiliated Hospital of Anhui Medical University, Hefei, 230022 Anhui Province China; 2 Department of Orthopedics, the First Affiliated Hospital of Anhui Medical University, No. 218, Jixi Road, Hefei, 230022 Anhui Province China

**Keywords:** USP1, Nonunion, Osteogenic differentiation, PI3K/Akt

## Abstract

**Background:**

Nonunion is a failure of fracture healing and a major complication after fractures. Ubiquitin-specific protease 1 (USP1) is a deubiquitinase that involved in cell differentiation and cell response to DNA damage. Herein we investigated the expression, function and mechanism of USP1 in nonunion.

**Methods and results:**

Clinical samples were used to detect the USP1 expression in nonunion. ML323 was selected to inhibit USP1 expression throughout the study. Rat models and mouse embryonic osteoblasts cells (MC3T3-E1) were used to investigate the effects of USP1 inhibition on fracture healing and osteogenesis in vivo and in vitro, respectively. Histological changes were examined by micro-computerized tomography (Micro-CT), hematoxylin & eosin (H&E) staining and Masson staining. Alkaline phosphatase (ALP) activity detection and alizarin red staining were used for osteogenic differentiation observation. The expression of related factors was detected by quantitative real-time PCR, western blot or immunohistochemistry (IHC). It was shown that USP1 was highly expressed in nonunion patients and nonunion rats. USP1 inhibition by ML323 promoted fracture healing in nonunion rats and facilitated the expression of osteogenesis-related factors and the signaling of PI3K/Akt pathway. In addition, USP1 inhibition accelerated osteogenic differentiation and promoting PI3K/Akt signaling in MC3T3-E1 cells.

**Conclusions:**

USP1 inhibition plays a promotive role in coordinating osteogenic differentiation and fracture healing during nonunion. PI3K/Akt may be the downstream pathway of USP1.

## Introduction

Bone is one of the most important organs in humans and animals, and is a tissue that can continuously remodel throughout the life. The formation of bone is a complex dynamic process, which is regulated by various bone growth factors [1]. Osteogenesis is a sequential cascade that pluripotent mesenchymal stem cells develop into osteoblasts, which then control the synthesis, secretion and mineralization of bone matrix [2, 3].

Bone nonunion, also called fracture nonunion, is a serious complication of fracture. The US Food and Drug Administration (FDA) defines nonunion as a fracture that fails to heal within nine months and shows no signs of healing for three consecutive months [4]. The probability of fracture nonunion occurrence is about 5% to 10% and fracture nonunion often causes pain, functional and psychological disorders for patients [5, 6]. Moreover, fracture nonunion results in poor quality of life, significant medical costs due to prolonged hospital stay and reoperation, even increased risk of death [7, 8]. The risk of fracture nonunion is associated with multiple factors, including fracture severity, fracture location, infection, comorbidities and medications [5, 9]. At present, surgery is the main treatment for fracture nonunion, but it remains a challenge in clinical practice because the etiology and mechanism of fracture nonunion are not clear. Searching for targets to promote fracture healing is one of the current research priorities.

Ubiquitin-specific peptidase 1 (USP1) is one of the members of ubiquitin-specific protease (USP) family, and is involved in cell differentiation and regulates cell response to DNA damage [10]. It has been shown that USP1 could form a deubiquitinase complex with USP1-associated factor 1 (UAF1) [11], and they participate in the pathogenesis of tumors and enhance the antiviral response together [12]. In addition, the function of the USP1/UAF1 complex in inflammation was discovered through its interaction with NLRP3 [13]. However, the function of USP1 in fracture healing has not been reported.

Bone formation is divided into intramembranous ossification and endochondral ossification [14]. Fracture healing often begins with the chemotaxis and proliferation of mesenchymal stem cells. Mouse embryonic osteoblast cell line MC3T3-E1 has high proliferation and differentiation characteristics. Hence, it is a classic model for studying osteogenic differentiation in vitro [15]. Studies have shown that promoting osteogenic differentiation in MC3T3-E1 cells is beneficial to fracture healing in nonunion rats [16, 17]. Importantly, in the process of osteogenic differentiation, USP1 modification significantly affected the osteogenic differentiation of osteoblasts [18]. As a selective inhibitor of USP1, ML323 has been reported to enhance the osteogenesis ability of the dental pulp stem cells [19]. Based on these facts, we hypothesized that inhibition of USP1 by ML323 would benefit fracture healing.

Phosphoinositide 3‐kinase (PI3K) is a member of heterodimeric lipid kinases, playing a key role in cellular activities. Akt is a kind of serine protein kinase served as the downstream target of PI3K [20]. The PI3K/Akt pathway has a great impact on the regulation of metabolism, gene expression, protein synthesis, cell proliferation and survival [21]. Activation of PI3K/Akt signaling pathway contributes to osteogenic differentiation and osteogenesis [22, 23]. The results of the study by Dana Goldbraikh et al. showed that USP1 promoted the deubiquitination of Akt and reduced its phosphorylation level, while inhibition of USP1 promoted the activation of PI3K/Akt pathway [24]. In this study, we also investigated the involvement of PI3K-Akt signaling in the function of USP1 in nonunion.

## Materials and methods

### Patient recruitment and sample tissue collection

This work was performed at the First Affiliated Hospital of Anhui Medical University, and it received the institutional ethics committee approval and followed the principles of the Declaration of Helsinki. Study participants were patients with atrophic nonunion of limbs long bone (*n* = 10) and patients with normal fracture healing (*n* = 9). Among the 10 nonunion patients (median age: 43, range 16–71, 7 males and 3 females), fracture site was mostly at the tibia, and most of these patients had closed nonunion. In nine patients with normal fracture healing (median age: 48, range 31–69, 8 males and 1 female), fracture site was mostly at the fibula. None of the above patients had a past medical history. All patients signed informed consent and consent for publication. Nonunion tissues from patients with nonunion and callus tissues from patients with normal fracture healing were collected by surgery for detection of target gene expression at the clinical level.

### Quantitative real-time polymerase chain reaction (qPCR)

Total RNA was extracted by the TRIpure lysate (BioTeke, Beijing). The concentration of RNA was determined by UV spectrophotometer (Thermo, MA). Reverse transcription was performed by using BeyoRT II M-MLV reverse transcriptase (Beyotime, Shanghai). Quantitative real-time PCR was performed by 2 × Taq PCR Master Mix on Exicycler 96 fluorescence quantitative PCR instrument (BIONEER, Daejeon). All gene expression was analyzed using the 2^–Δ∆Ct^ method. The primer sequences were as follows: rat USP1: forward, GTGGCTTGGAGTTTGATT and reverse, CATTAGTCGGCTTTGTGC; homo USP1: forward, TATTTGCGGTTGTGATG and reverse, CAATGGTTCTGGCTTAC.

### Nonunion model and treatment

According to the methodology of previous literature [25], 12-week-old female Wistar rats (purchased from Liaoning Changsheng biotechnology co., Ltd, Benxi) were selected and randomly divided into following groups: fracture healing group, nonunion group and nonunion + ML323 group. Under anesthesia, the lateral skin of the right thigh of rats was dissected in a sterile environment. The right femur was exposed after blunt dissection of the muscle, and a transverse osteotomy was performed. The periosteum within 2 mm of the broken end of nonunion group was burned with a heated needle, and care was taken to avoid burning the cortex of the bone. A 1.5-mm-diameter, 45-mm-length medical stainless needle was inserted through the medullary cavity of the distal femur. Then, the proximal end of the needle was threaded through the top of the greater trochanter of the femur while bending the proximal end to prevent detachment. During the fixation of femoral fractures in rats, a spacer is placed at the fracture site to maintain a consistent fracture gap length (approximately 1.5 mm). Finally, the lateral femoral incision was sutured layer-by-layer. The periosteum in fracture healing group was not burned while the rest procedures were consistent. Rats in nonunion + ML323 group received intraperitoneal injection of 3 mg/kg ML323 (Aladdin, Shanghai) once a week for six times. Rats in nonunion group were given the same amount of solvent (3 mg/kg). The fracture tissues was collected after 6 weeks of model establishment. Part of the fracture tissues were fixed with 4% paraformaldehyde, the other part of the fracture tissues was frozen in liquid nitrogen and stored in – 70 °C ultra-low temperature refrigerator for subsequent experiments. These experiments were performed in strict accordance with the Guideline for the Care and Use of Laboratory Animals and approved by the Experimental Animals Ethics Committee of Anhui Medical University.

### Western blot

Total protein from fracture tissues was extracted from all groups six weeks after the operation, followed by measuring concentration of protein extract with a BCA kit (Beyotime, Shanghai). Equal amounts of protein samples were subjected to sodium dodecyl sulfate polyacrylamide gel (SDS-PAGE) electrophoresis and transferred to polyvinylidene fluoride (PVDF, Millipore, MA) membrane. The obtained PVDF membrane was incubated overnight with primary antibodies against USP1 (1:3000, Proteintech, Wuhan), BMP2 (1:1000, Affinity, Changzhou), RUNX2 (1:1000, Abclonal, Wuhan), OCN (1:500, Abclonal, Wuhan), Akt (1:1000, Affinity, Changzhou), p-Akt^T308^ (1:1000, Affinity, Changzhou) and p-Akt^S473^ (1:1000, Affinity, Changzhou) at 4 °C. At last, after washed by TBST (Tris-buffered saline + Tween), the membranes were incubated with secondary antibodies goat anti-rabbit IgG (Abclonal, Changzhou) and goat anti-mouse IgG (Abclonal, Wuhan) at 37 °C for 40 min. The bands were detected by enhanced chemiluminescence (ECL, Beyotime, Shanghai) detection reagent and the optical density values of the target bands were analyzed by image analysis software (Tanon, Shanghai).

### Immunohistochemistry (IHC) and hematoxylin & eosin (H&E) staining

The right femur was collected and fixed in 4% paraformaldehyde for 48 h, decalcified with ethylenediaminetetraacetic acid (EDTA) decalcium solution at 37 °C and embedded in paraffin. Sections with 5-µm thickness were obtained, deparaffinized to water, and then subjected to histological staining. For IHC, the sections were placed in boiled antigen repair solution for 10 min and immersed in PBS for 5 min in sequence, so as to repeat three times for antigen repair. Next, the sections were incubated with 3% H_2_O_2_ (Sinopharm, Shanghai) for 15 min at room temperature and immersed in PBS for 5 min. After three repetitions, the sections were blocked with 1% BSA (Sangon, Shanghai) and incubated with primary antibodies targeting USP1 (1:200, Proteintech, Wuhan) and BMP2 (1:200, Affinity, Liyang) overnight at 4 °C. Then, they were incubated with secondary antibodies HRP labeled goat anti-mouse or anti-rabbit IgG (1:500, ThermoFisher, Waltham) for 60 min at 37 °C. Finally, DAB (Maxim, Fuzhou) was used to display the color of the sections, and the sections were counterstained with hematoxylin (Solarbio, Beijing) and observed under a microscope (Olympus, Tokyo). For H&E staining, the sections were successively immersed in hematoxylin (Solarbio, Beijing) for 5 min, distilled water for 5 min, and 1% hydrochloric acid alcohol for 3 s, flushed with running water for 20 min, immersed in distilled water for 2 min, and finally immersed in eosin (Sangon, Shanghai) staining solution for 3 min. After the sections were blocked, the sections were viewed with a microscope (Olympus, Tokyo) and photographed.

### Micro-computerized tomography (Micro-CT)

Rats were imaged using a high-resolution micro-computerized tomography (Quantum GX μCT System, PerkinElmer, Waltham) at weeks 6. Visualization and the data of bone volume/total volume × 100% (BV/TV), trabecular number (TbN), trabecular thickness (Tb.Th) and trabecular separation (Tb.Sp) were obtained using the Quantum GX μCT Workstation imaging software (PerkinElmer, Waltham).

### Masson staining (MS)

The prepared paraffin sections were first nucleated with Regaud hematoxylin staining solution, then stained with Lichun red acid magenta dye (Sinopharm, Shanghai), and finally counterstained with aniline blue. The blue color was observed using an Olympus BX53 microscope (Tokyo), which indicates new or mature bone.

### Cell culture

MC3T3-E1 cells were purchased from iCell Bioscience Inc (Shanghai) and were incubated in a 5% CO_2_ incubator at 37 ℃ overnight. Cells were divided into following groups: control group, osteogenic differentiation medium (ODM) group, ODM + Vehicle group and ODM + ML323 group. In ODM group, MC3T3-E1 cells were cultured in minimum essential medium α (MEMα, iCell bioscience, Shanghai), a kind of ODM containing 10% fetal bovine serum (FBS, Zhejiang TianHang Biotechnology Co. Ltd., Huzhou), 10 mM β-glycerophosphate (Macklin, Shanghai) and 50 µg/mL ascorbic acid (Aladdin, Shanghai) in a 5% CO_2_ incubator at 37 ℃ to induce osteogenic differentiation for up to 14 days. Medium of ODM + ML323 group and ODM + Vehicle group were supplemented with ML323 (Macklin, Shanghai) and equal volume of solvent, respectively.

### Alkaline phosphatase (ALP) activity measurement

The cell samples of each group were resuspended with PBS, and the cells were broken by ultrasonic broken instrument (Ningbo Scientz Biotechnology Co.,Ltd., Ningbo) under the condition of ice bath on differentiation day 7. The protein content of the cell suspension was determined by BCA assay kit (Solarbio, Beijing). For quantification of ALP activity, ALP kit (Nanjing Jiancheng, Nanjing) was used.

### Alizarin red staining and quantification

On osteogenic differentiation day 14, cell culture medium was discarded from the cell suspension of each group, and after washing twice with PBS, the cells were stained with alizarin red (Yuanye Bio, Shanghai) for 20 min. After washing with PBS twice again, photos were taken under a microscope (Olympus, Tokyo). For quantification of calcium deposition, the alizarin staining was extracted using 10% cetylpyridinium chloride (Macklin, Shanghai). Then, the absorbance of the extract was measured at 570 nm.

### Statistical analysis

All data were presented as mean ± standard deviation. Statistical tests were performed with Prism 8.0.2 (GraphPad, La Jolla). Comparisons of two groups were using unpaired Student’s t test and comparisons of multiple groups were assessed using one-way analysis of variance (ANOVA). Significance levels are marked as # or *p < 0.05, ## or **p < 0.01 and ns not significant.

## Results

### USP1 was highly expressed in nonunion patients and rats

**Fig. 1:**
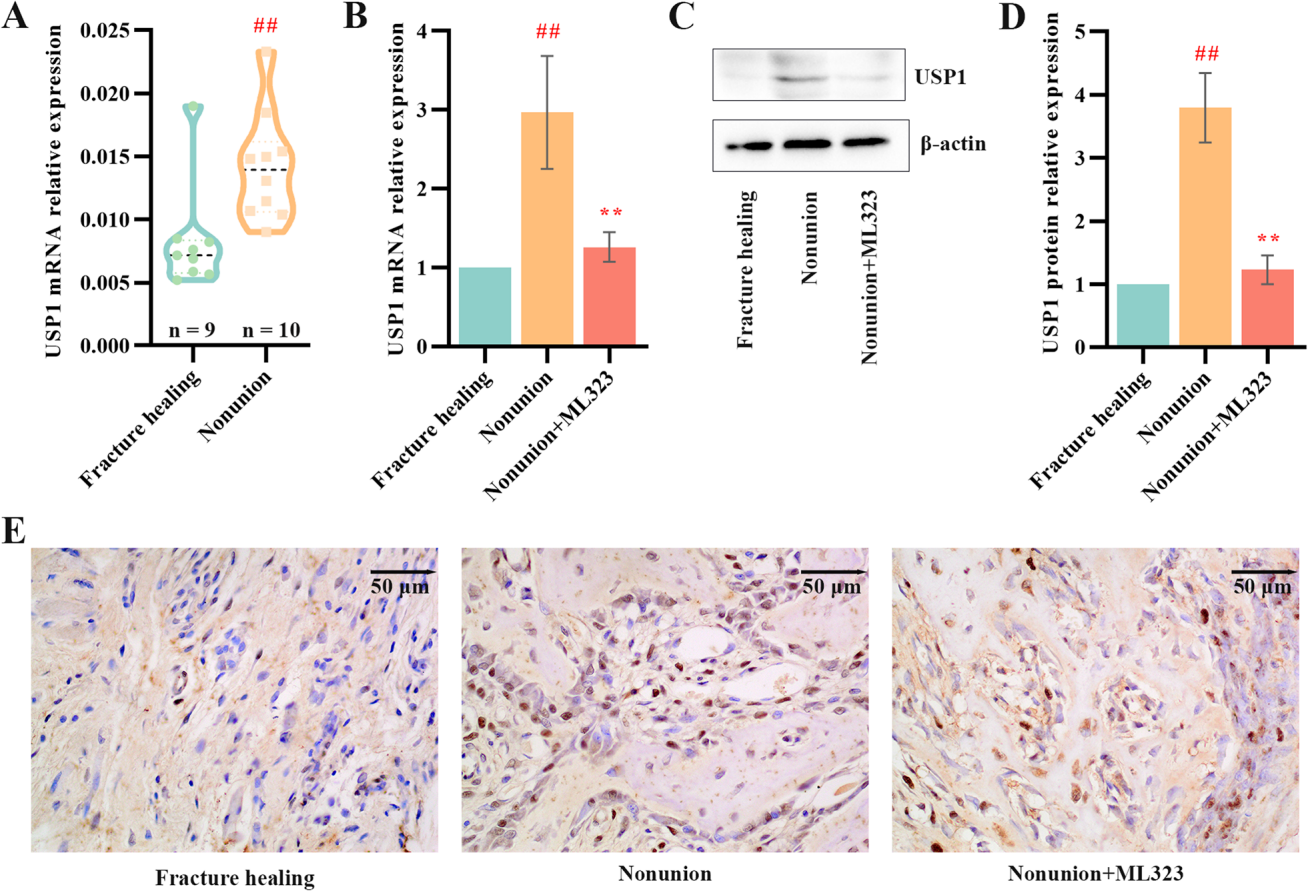
High expression of USP1 in nonunion tissues of patients and rats. **A** Relative mRNA expression of USP1 in fracture healing (*n* = 9) and nonunion patients (*n* = 10). **B** Relative mRNA expression of USP1 in rats of fracture healing group, nonunion group and nonunion + ML323 group (nonunion rats treated with ML323) (*n* = 5/group). **C** & **D** Immunoblot and quantitative analysis for USP1 in rats of fracture healing group, nonunion group and nonunion + ML323 group (nonunion rats treated with ML323) (n = 5/group). β-actin was used as a loading control. **E** Representative immunohistochemical (IHC) staining images of the expression and localization of USP1 in rats of fracture healing group, nonunion group and nonunion + ML323 group (nonunion rats treated with ML323). Scale bar = 50 µm. ## indicates *p* < 0.01 in comparison with the fracture healing group; ** indicates *p* < 0.01 in comparison with the nonunion group

To validate the USP1 expression in nonunion patients, we performed qPCR to analyze the mRNA expression levels of USP1 in fracture healing and nonunion samples. The expression levels of USP1 were higher in nonunion tissues than those in fracture healing tissues (Fig. 1A). In a rat model of nonunion, intraperitoneal injection of ML323 was for suppression of USP1 expression. The mRNA and protein expression levels of USP1 were higher in nonunion tissues of rats while the expression of USP1 was successfully suppressed in nonunion + ML323 group (Fig. 1B–D). We also confirmed these results by IHC staining assay that USP1 was highly expressed in nonunion tissues of rats than that in fracture healing, and that ML323 effectively inhibited the expression of USP1 in nonunion rats (Fig. 1E).

### USP1 inhibition promoted fracture healing in nonunion rats.

**Fig. 2:**
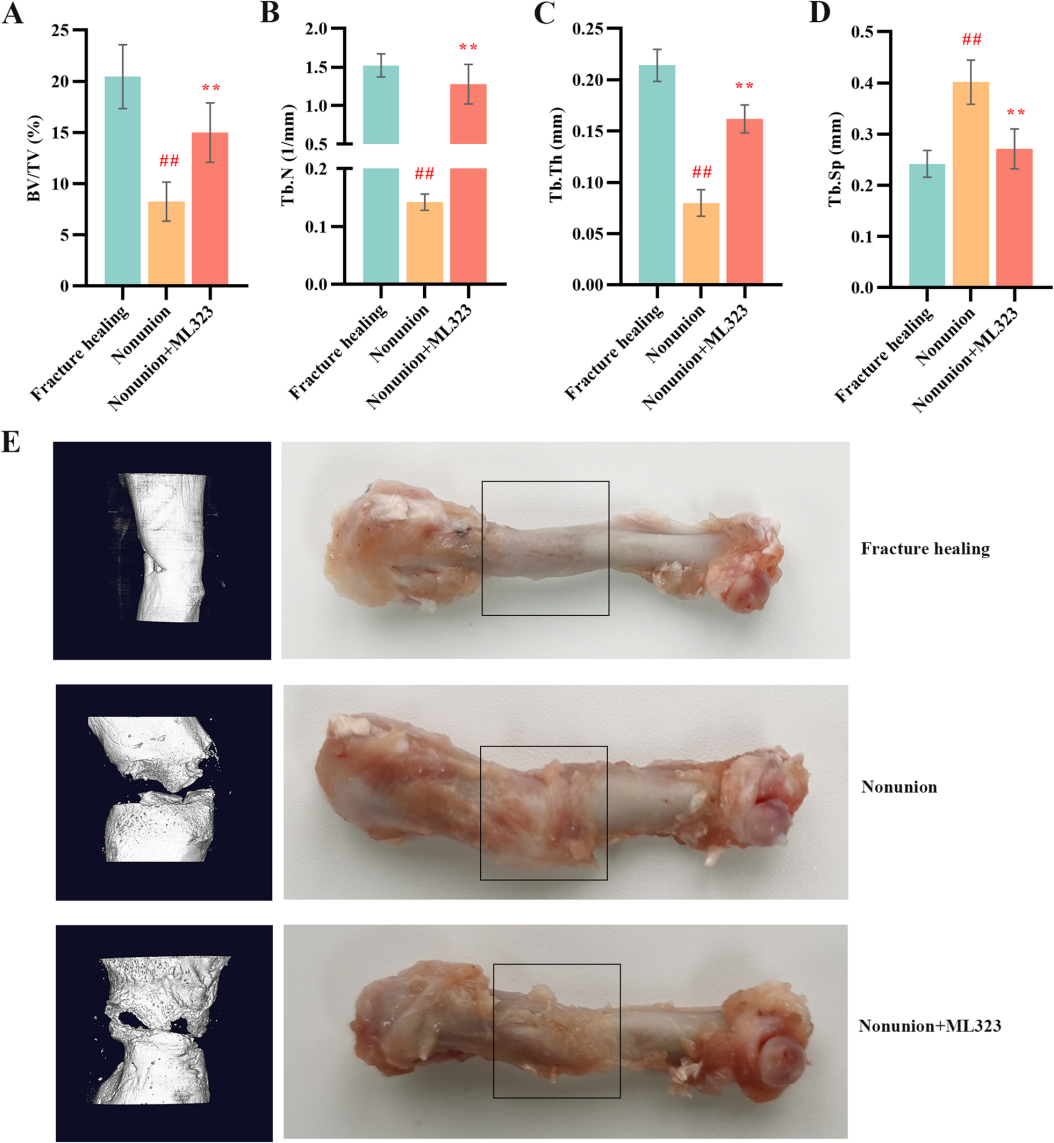
USP1 inhibition by ML323 promoted bone morphological changes in rats. **A**–**D** The BV/TV, Tb.N, Tb.Th and Tb.Sp of right femur were measured using micro-CT (micro-computerized tomography) in rats of fracture healing group, nonunion group and nonunion + ML323 group (nonunion rats treated with ML323) (*n* = 5/group). BV/TV, bone volume/total volume × 100%; Tb.N, trabecular number; Tb.Th, trabecular thickness; Tb.Sp, trabecular separation. **E** The micro-CT images (left) and typical morphology images of right femur (right) in rats of fracture healing group, nonunion group and nonunion + ML323 group (nonunion rats treated with ML323). ## indicates *p* < 0.01 in comparison with the fracture healing group; ** indicates *p* < 0.01 in comparison with the nonunion group

**Fig. 3:**
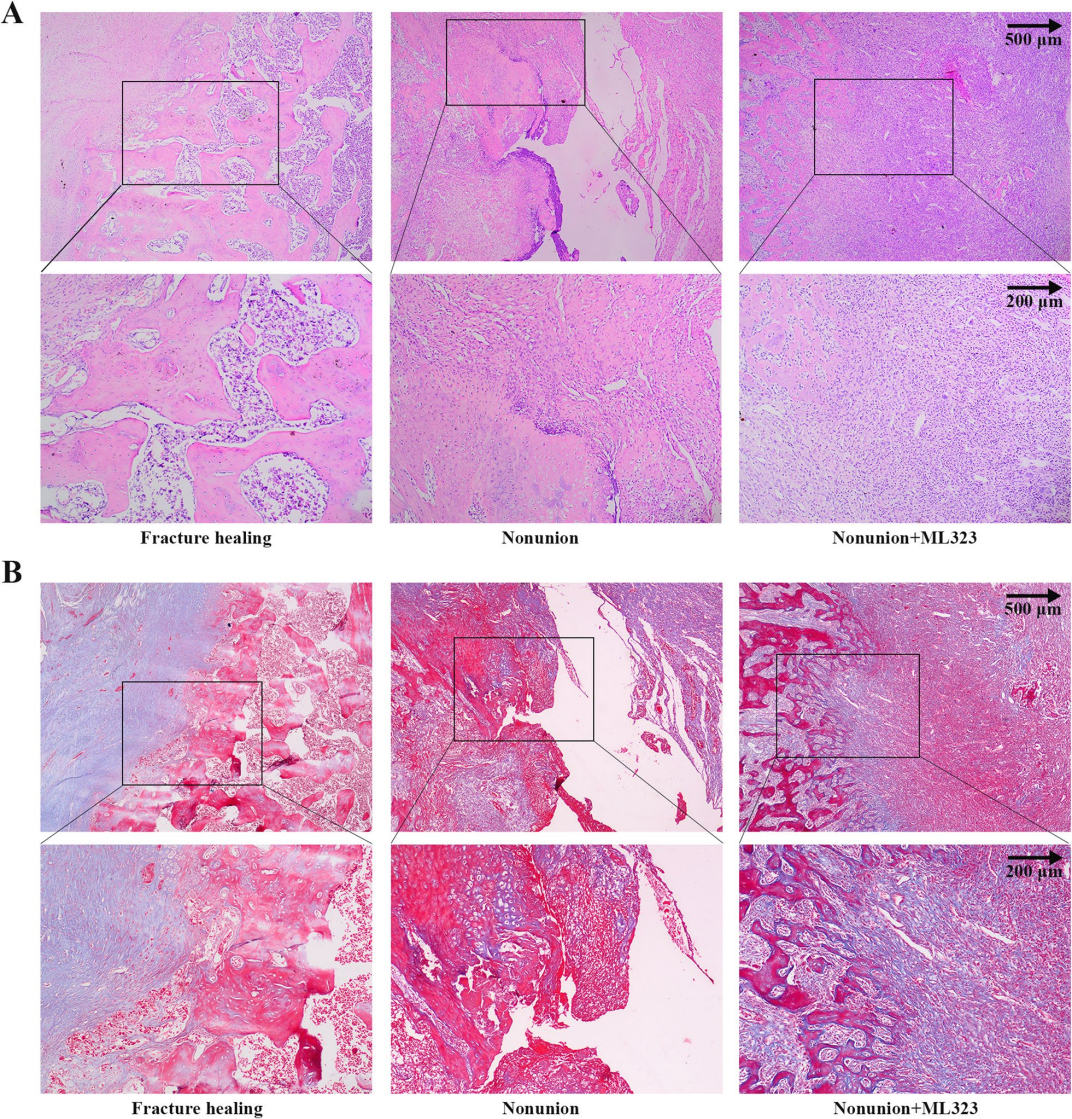
USP1 inhibition by ML323 enhanced bone regeneration in rats. **A** & **B** Hematoxylin and eosin (**H** & **E**) and Masson (MS) staining images of right femur tissue sections in rats of fracture healing group, nonunion group and nonunion + ML323 group (nonunion rats treated with ML323). Scale bar = 500 µm (above) and scale bar = 200 µm (below) in the magnified images

To evaluate the effect of USP1 inhibition on fracture healing in nonunion rats, micro-CT and histological staining were performed. Micro-CT analysis of trabecular bone from the right femur revealed that the BV/TV, Tb.N and Tb.Th were significantly increased and the Tb.Sp was significantly decreased when USP1 inhibition compared to nonunion group (Fig. 2A–D). The nonunion group showed the opposite performance compared to the fracture healing group. Micro-CT images of the fracture site and typical morphology images of right femur were shown in Fig. 2E. H&E staining results showed that there were medulla spaces with mesenchymal cells in fracture healing group, while in the nonunion group, fibrotic tissue formation was observed (Fig. 3A). In addition, fibrotic tissue was reduced in nonunion + ML323 group. The quality of bone repair was further evaluated by Masson staining (Fig. 3B). Compared with the fracture healing group, the nonunion group had less bone formation and poor bone quality. Moreover, MS sections of nonunion + ML323 group showed more blue staining, indicating good bone repair.

### USP1 inhibition facilitated the expression of osteogenesis-related factors and the signaling of PI3K/Akt pathway in nonunion rats.

**Fig. 4:**
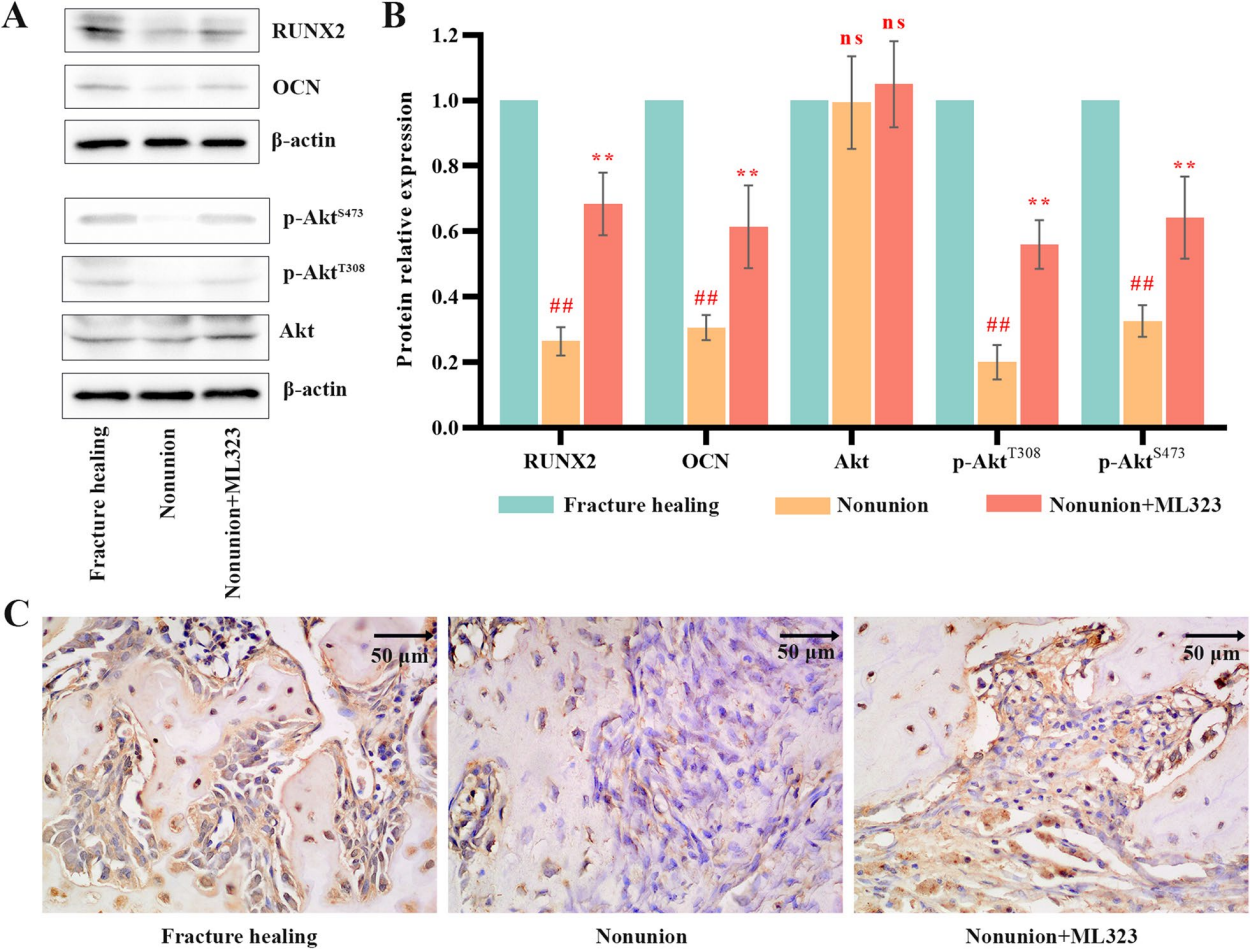
USP1 inhibition increased the levels of osteogenesis-related factors and the phosphorylation of PI3K/Akt pathway in nonunion rats. **A** & **B** Immunoblot and quantitative analysis for RUNX2, OCN, Akt, p-Akt^T308^ and p-Akt^S473^ in rats of fracture healing group, nonunion group and nonunion + ML323 group (nonunion rats treated with ML323) (*n* = 5/group). β-actin was used as a loading control. Akt was used as an internal control for p-Akt^T308^ and p-Akt^S473^. **C** IHC staining images of the expression of BMP2 in rats of fracture healing group, nonunion group and nonunion + ML323 group (nonunion rats treated with ML323). Scale bar = 50 µm. ## indicates *p* < 0.01 in comparison with the fracture healing group; ** indicates *p* < 0.01 in comparison with the nonunion group and ns indicates not significant

Immunoblot and quantitative analysis (Fig. 4A and B) showed that RUNX2 and OCN were significantly increased when USP1 inhibition. Although inhibition of USP1 had no obvious effect on Akt phosphorylation, it enhanced Akt phosphorylation at T308 and S473. The expression of BMP2 was further detected by IHC staining (Fig. 4C). Results showed that BMP2 was highly expressed in the fracture healing group and the nonunion + ML323 groups, but lowly expressed in the nonunion group.

### USP1 inhibition accelerated osteogenic differentiation and increasing PI3K/Akt signaling in MC3T3-E1 cells

**Fig. 5:**
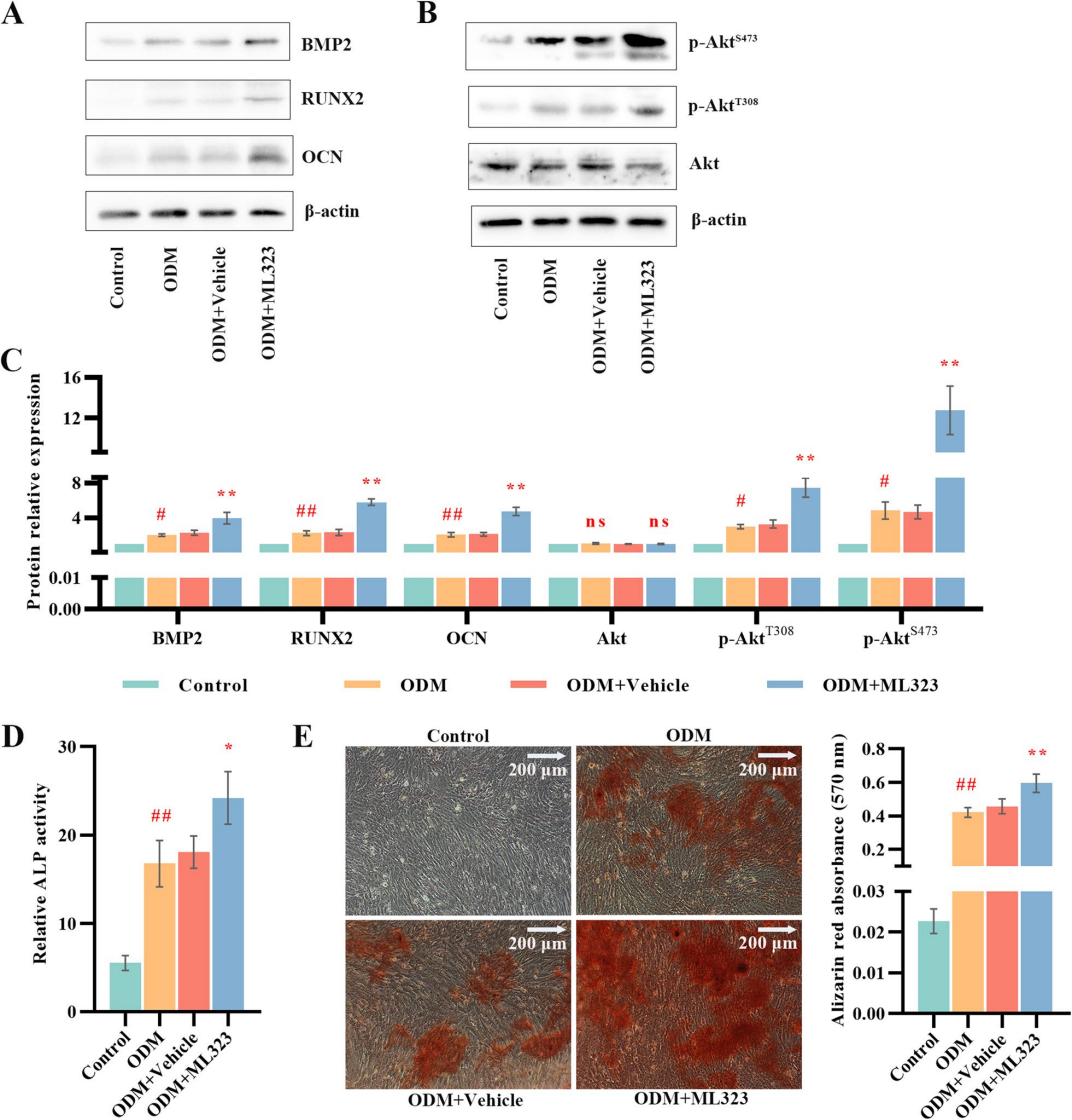
USP1 inhibition promoted osteogenic differentiation of MC3T3-E1 and PI3K/Akt pathway. **A**–**C** Protein levels and quantitative analysis for BMP2, RUNX2, OCN, p-Akt^S473^, p-Akt^T308^ and Akt in control group, ODM group, ODM + Vehicle group and ODM + ML323 group after osteogenic differentiation was induced for 7 days (*n* = 3/group). β-actin was used as a loading control. Akt was used as an internal control for p-Akt^T308^ and p-Akt^S473^. **D** Quantitative measurement of ALP activity in each group after osteogenic differentiation was induced for 7 days (*n* = 3/group). **E** Alizarin red staining (left) and quantification (right) were performed on day 14 of osteogenic differentiation (*n* = 3/group). ODM, osteogenic differentiation medium. Scale bar = 200 µm. # and ## indicate *p* < 0.05 and *p* < 0.01 in comparison with the control group, respectively; * and ** indicate *p* < 0.05 and *p* < 0.01 in comparison with the ODM + Vehicle group, respectively; ns indicates not significant

To determine the role of USP1 in osteogenic differentiation, MC3T3-E1 cells were cultured and induced to osteogenic differentiation. Analysis of osteogenesis-related factors after induction of osteoblastic differentiation for seven days showed significant changes in BMP2, RUNX2 and OCN protein levels in both osteogenic differentiation medium (ODM) group and ODM + ML323 group compared to control group and ODM + Vehicle group, respectively (Fig. 5A–C). We also found an increase in the levels of Akt^T308^ and Akt^S473^ phosphorylation; however, no changes in Akt phosphorylation were detected. ALP activity was further examined after osteogenic differentiation was induced for 7 days and results showed that ALP activity of cells was significantly increased in ODM + ML323 group (Fig. 5D). Likewise, quantitative of Alizarin Red staining, upon 14-day osteoblastic induction, was significantly higher in cells from ODM + ML323 group (Fig. 5E). These results concluded that USP1 inhibition positively impacted osteogenic differentiation in the MC3T3-E1 cells.

## Discussion

Nonunion brings a lot of physical and psychological troubles to patients, making it particularly important to study the pathogenesis of fracture nonunion. There are many factors affecting fracture nonunion, including mechanical, biological, patient-dependent and patient-independent factors [26]. Although there have been many theories and studies on nonunion [27–29], the mechanisms are still poorly understood. Waki et al. [30] reported that the functions of certain miRNAs and changes to their patterns of expression are crucial in the pathogenesis of nonunion. In addition, USP1 was reported to be a direct target of miR-192-5p in osteosarcoma cells [31]. At present, studies on USP1 mainly focus on cancer and tumors [32, 33], while its role in nonunion is still unknown. Some members of the USP family have been shown to promote or inhibit osteogenic differentiation in a recent review [34]. In this study, our clinical findings confirmed that USP1 mRNA levels were elevated in nonunion tissues of patients with atrophic nonunion of limbs long bone.

In order to explore the expression and function of USP1 in nonunion, animal models of nonunion were established. In nonunion rats, both the mRNA and protein expression of USP1 were upregulated, whereas suppressed after intraperitoneal injection of ML323 in nonunion rats. ML323 is a small molecule effective inhibitor of USP1, and it targets two major DNA damage response pathways by inhibiting USP1 simultaneously [35]. Baozhi Song et al. first found that USP1 is a key downstream target gene of ML323 through GSEA analysis, and inhibition of USP1 restrained cell proliferation and affected cell progression of ovarian cancer [33]. Moreover, previous studies have shown that USP1 was expressed at high levels in malignant diseases such as osteosarcoma [18], human breast cancer [36] and hepatocellular carcinoma [37]. USP4 which together with USP1 belongs to the USP family, negatively regulates osteoblast differentiation and bone formation induced by WNT [38]. However, USP26 has been shown osteoprotective role of regulating bone homeostasis by coordinating bone formation and resorption [39]. In addition, osteogenic differentiation was inhibited by USP34 depletion [40] and USP2 was also reported to be associated with osteogenesis [41]. These evidences suggest that USP family is essential for osteogenic differentiation and bone formation. Therefore, we further explored the effect of USP1 inhibition on fracture healing in nonunion rats.

The results of micro-CT of the right femur from nonunion rats demonstrated that ML323 treatment improved the microstructure of the trabecular bone. Increases in BV/TV, Tb.N, Tb.Th and decreases in Tb.Sp of right femur were found in the nonunion rats receiving ML323, which was confirmed by results of histomorphology. Similar dynamics of bone neogenesis have previously been described in rat models [42, 43]. From a molecular perspective, bone healing is characterized by high expression of growth factors involved in the proliferation and differentiation of fibroblasts, osteoblasts, and endothelial cells [44, 45]. RUNX family transcription factor 2 (RUNX2), an important member of the Runx TF family, is one of the important factors controlling osteoblast development and bone formation [46]. Bone morphogenetic protein 2(BMP-2) is considered to be the most effective bone repair cytokine [47]. Osteocalcin (OCN) is an important part of the extracellular matrix that acts as a late osteogenic marker [48, 49]. In our study, USP1 inhibition facilitated the expression of these osteogenesis-related factors. In Yanping Gong et al. study, increased expression of osteogenic markers was used to explain the possible mechanism by which adiponectin promotes fracture healing [50].

The PI3K/Akt signaling pathway is involved in a wide range of physiological processes. A previous report showed that downregulation of USP1 increased p-Akt^T308^ levels in muscle of fasting mice with little effect on p-Akt^S473^ levels [24], while another report showed that downregulation of USP1 significantly increased p-Akt^S473^ levels and slightly increased p-Akt^T308^ levels in lung cancer cells [51]. The results of this study showed that USP1 inhibition significantly increased p-Akt^S473^ and p-Akt^T308^ levels, slightly increased Akt level. Inversely, human differentiated embryonic chondrocyte expressed gene 1 deficiency attenuates the PI3KCA/Akt/GSK3β signaling by affecting levels of PI3KCA, p-Ser473-Akt and p-Ser9-GSK3β, which has a negative effect on osteogenic differentiation in the bone mesenchymal stem cells [52]. In addition, overexpression of miR-181a/b-1 increases PI3K/Akt signaling by inducing p-Akt^T308^ phosphorylation during osteogenesis [53]. Therefore, we verified the role of USP1 inhibition in the differentiation of MC3T3-E1 osteoblasts and its effect on the PI3K/Akt pathway. As expected, inhibition of USP1 may enhance the osteogenic differentiation of MC3T3-E1 in vitro by activating PI3K/Akt signaling pathway, which were consistent with previous studies [54–56]. Measurement of ALP activity and quantification of calcification deposits in extracellular matrix are important means to evaluate ossification and mineralization of osteoblasts [55]. In the present study, our results further demonstrated that inhibition of USP1 contributed to increase of ALP activity and mineralization in osteoblasts. Interestingly, a recent study also suggested that ALP activity and alizarin red staining intensity were significantly decreased after USP34 depletion [40]. USP1 and USP6 inhibit osteogenesis in osseous tumors have been shown in previous studies [18, 57]. These data and our findings suggest that different members of the USP family have different roles in osteogenic differentiation and bone formation. Our study mainly demonstrated the expression and function of USP1 in nonunion.

## Conclusion

In summary, our study shows that expression of USP1 in nonunion patients and rats increases and inhibition of USP1 promotes the fracture healing by facilitating the expression of osteogenesis-related factors and the signaling of PI3K/Akt pathway in nonunion rats. Moreover, inhibition of USP1 from MC3T3-E1 cells results in improvement of osteogenic differentiation and activation of PI3K/Akt pathway. Mechanically, PI3K/Akt may be the downstream pathway of USP1 inhibition. Our study provides a novel direction and demonstrates that USP1 represents a potential therapeutic target for nonunion treatment.

## Data Availability

The datasets used and analyzed during the current study are available from the corresponding author on reasonable request.

## Abbreviations

USP1: Ubiquitin-specific protease 1
Micro-CT: Micro-computerized tomography
H&E: Hematoxylin & eosin
ALP: Alkaline phosphatase
IHC: Immunohistochemistry
FDA: Food and drug administration
UAF1: USP1-associated factor 1
PI3K: Phosphoinositide 3‐kinase
qPCR: Quantitative real-time polymerase chain reaction
SDS-PAGE: Sodium dodecyl sulfate polyacrylamide gel
PVDF: Polyvinylidene fluoride
TBST: Tris-buffered saline + Tween
EDTA: Ethylenediaminetetraacetic acid
ECL: Enhanced chemiluminescence
BV/TV: Bone volume/total volume × 100%
TbN: Trabecular number
Tb.Th: Trabecular thickness
Tb.Sp: Trabecular separation
MS: Masson staining
ODM: Osteogenic differentiation medium
MEMα: Minimum essential medium α
FBS: Fetal bovine serum
RUNX2: RUNX family transcription factor 2
BMP-2: Bone morphogenetic protein 2
OCN: Osteocalcin
